# Explainable Artificial Intelligence for Prediction of Complete Surgical Cytoreduction in Advanced-Stage Epithelial Ovarian Cancer

**DOI:** 10.3390/jpm12040607

**Published:** 2022-04-10

**Authors:** Alexandros Laios, Evangelos Kalampokis, Racheal Johnson, Amudha Thangavelu, Constantine Tarabanis, David Nugent, Diederick De Jong

**Affiliations:** 1 Department of Gynaecologic Oncology, St James’s University Hospital, Leeds LS9 7TF, UK; racheal.johnson2@nhs.net (R.J.); amudhathangavelu@nhs.net (A.T.); david.nugent@nhs.net (D.N.); diederick.dejong@nhs.net (D.D.J.); 2 Department of Business Administration, University of Macedonia, 54636 Thessaloniki, Greece; ekal@uom.edu.gr; 3 Center for Research & Technology HELLAS (CERTH), 6th km Charilaou-Thermi Rd., 57001 Thessaloniki, Greece; 4 Department of Internal Medicine, School of Medicine, New York University, NYU, Langone Health, New York, NY 10016, USA; constantine.tarabanis@nyulangone.org

**Keywords:** Explainable Artificial Intelligence, complete cytoreduction, epithelial ovarian cancer

## Abstract

Complete surgical cytoreduction (R0 resection) is the single most important prognosticator in epithelial ovarian cancer (EOC). Explainable Artificial Intelligence (XAI) could clarify the influence of static and real-time features in the R0 resection prediction. We aimed to develop an AI-based predictive model for the R0 resection outcome, apply a methodology to explain the prediction, and evaluate the interpretability by analysing feature interactions. The retrospective cohort finally assessed 571 consecutive advanced-stage EOC patients who underwent cytoreductive surgery. An eXtreme Gradient Boosting (XGBoost) algorithm was employed to develop the predictive model including mostly patient- and surgery-specific variables. The Shapley Additive explanations (SHAP) framework was used to provide global and local explainability for the predictive model. The XGBoost accurately predicted R0 resection (area under curve [AUC] = 0.866; 95% confidence interval [CI] = 0.8–0.93). We identified “turning points” that increased the probability of complete cytoreduction including Intraoperative Mapping of Ovarian Cancer Score and Peritoneal Carcinomatosis Index < 4 and <5, respectively, followed by Surgical Complexity Score > 4, patient’s age < 60 years, and largest tumour bulk < 5 cm in a surgical environment of optimized infrastructural support. We demonstrated high model accuracy for the R0 resection prediction in EOC patients and provided novel global and local feature explainability that can be used for quality control and internal audit.

## 1. Introduction

Primary epithelial cancer of the fallopian tube, ovary, or peritoneum (EOC) is the deadliest gynaecological malignancy in the Western world [1]. Over 70% of women diagnosed with EOC have advanced disease at presentation (FIGO stage 3–4) [1]. Surgical cytoreduction combined with platinum-based chemotherapy, either as treatment following surgery (adjuvant, ACT) or as treatment both before and after surgery (neoadjuvant, NACT), has long been the cornerstone of EOC management [2,3]. The surgery aims for maximal cytoreduction of all visible disease, ideally reaching a total macroscopic tumour clearance (CC0). Survival outcomes are inversely related to the initial tumour load and the residual disease following cytoreductive surgery [4].

Landmark randomized studies demonstrated prognostic non-inferiority of NACT over primary debulking surgery (PDS) [5,6]. The NACT group displayed higher complete cytoreduction (CC0) rates compared with the PDS group, but these were lower compared to other studies [7]. If CC0 offers the best prognosis for EOC patients, surgeons should focus on CC0 as a primary outcome measure in both the PDS and IDS settings [8,9]. Such efforts frequently require extensive surgical procedures including multi-visceral resection techniques such as peritoneal stripping, diaphragmatic, splenic, liver, and gastrointestinal resections. The Aletti surgical complexity scoring system (SCS) was developed and validated to reflect surgical complexity, and to identify those patients at risk of severe morbidity [10].

In the realm of personalized medicine, innovative data mining technologies such as Artificial Intelligence (AI) can be used for monitoring quality improvement and delivery of modern ovarian cancer care. AI technology has been exponentially demonstrating its potential applications across various medical domains [11]. Examples include accurate classification between benign and malignant ovarian neoplasms using the CEA and HE4 biomarkers [12], development of an EOC-specific predictive framework for clinical staging, disease burden, and prognosis based on multiple blood biomarkers [13]. We previously identified an AI method, the k-NN model, which is very much reflective of ‘previous clinical experience’ as a promising and versatile tool for CC0 prediction in serous EOC [14]. Consequently, we investigated the problem of EOC survival, and highlighted the importance of AI-based feature selection for two-year prognosis prediction with satisfactory accuracy [15].

At the same time, explainability methods are required to render AI more attractive to clinicians and patients. Despite the recent growth of AI systems in medicine, explainable models remain scarce with only few examples in the fields of oncology [16]. This lack of decipherable AI has created doubt regarding its validity within clinical practice. Explainable AI (XAI) techniques have been recently introduced to explain specific decisions made by AI models, while maintaining a high level of learning performance [17]. As expected, the development of XAI in medical applications is in its infancy [18]. Currently, the definition of complete cytoreduction merely indicates the absence of macroscopically visible residual tumour cells in the tumour bed after surgical cytoreduction. In reality, the surgeons consider themselves responsible not only for the resection of the large bulk of primary cancer, but also for dealing with microscopic residual disease [19]. Unfortunately, R0 resections are not always realized in the complex environment of the operating room, and many surgeons seek objective strategies to evaluate the outcomes of their cytoreduction. This can be used not only for personalised planning of surgical strategy and subsequent treatment for the patient, but for self-assessment and improvement of clinicians’ individual judgment. Moreover, although previous complex AI approaches provide good prediction accuracy, their application in a clinical setting is limited because their predictions are difficult to interpret, and hence not actionable. Explainable Artificial Intelligence would help augment CC0 prediction for quality control and internal audit purposes. The objective of this study was to develop a predictive model for CC0 resection, and to support the explainability of the prediction as a binary classification problem, using the prospectively registered data of EOC patients who underwent surgical cytoreduction. The primary outcomes were the development and performance of the predictive AI model for CC0, application of methodology to explain the prediction, and evaluation of interpretability by analysing feature interactions. The secondary outcome was the exploration of potential clinical implementation of the AI model.

## 2. Materials and Methods

Prospective registered data in the hospital-wide Patient Pathway Manager (PPM) database from 576 consecutive women with EOC, who underwent cytoreductive surgery with the intention to be treated at St James’s University Hospital, Leeds, by a certified gynaecological oncology surgeon from January 2014 to December 2019 were analysed. This database was developed internally for clinical trials and integrated with an electronic patient record system [20]. Our hospital is a tertiary centre, recently accredited by the European Society of Gynaecological Oncology (ESGO) as a centre of excellence for ovarian cancer surgery. Tumour staging was reported by the 2014 International Federation of Gynaecology and Obstetrics (FIGO) classification [21].

In a personalized protocol, women underwent either PDS or three to four cycles of NACT followed by IDS if they had: FIGO stage 4 disease; poor performance status (PS); uncertainty about the possibility of optimal tumour removal. Only EOC patients with at least one pre-treatment CA125 value were included in the study. Excluded were patients aged < 18 years, as well as those with non-epithelial histology, synchronous primary malignancy, and those undergoing emergency surgery for bowel obstruction by general surgeons. Patients who had progressive disease following three courses of NACT were excluded from the analysis. Patients with low-grade EOC were offered NACT, but were counselled regarding the chemo-resistant nature of the disease, and therefore the limited lack of efficacy. The study was conducted according to the guidelines of the Declaration of Helsinki and approved by the Leeds Teaching Hospitals Trust Institutional Review Board (MO20/133163/18.06.20). Informed written consent was obtained from all patients. Formal case discussion at the central gynaecological oncology multidisciplinary team (MDT) meeting was undertaken for all patients prior to treatment initiation.

All patients underwent the standard institutional therapy for ovarian cancer, namely primary surgical cytoreduction, which involved explorative laparotomy, abdominal hysterectomy with bilateral salpingo-oophorectomy omentectomy, and peritoneal sampling. Additional surgical procedures were performed according to practice recommendations from the British Gynaecological Cancer Society (BGCS) to improve cytoreduction rates [22]. Laparoscopic assessment of disease respectability prior to embarking on surgical cytoreduction is not standard practice in our institution. It is infrequently used upon the MDT’s recommendation. Following implementation of the Enhanced Recovery After Surgery (ERAS) pathway [23] in late 2015, a paradigm shift towards more complex multi-visceral surgery was initiated in the years 2016 and 2017. This was facilitated by the appointment of new Gynaecological Oncology Consultant colleagues with specific training in (ultra-) radical surgery for EOC; the development of governance models to support patient safety when undergoing maximal effort cytoreductive surgery for EOC by joint working for gynaecologic oncologists and surgeons from other disciplines; further and more robust optimisation of the ERAS protocols with the appointment of specialized enhanced recovery nurses; expansion of the availability of critical care unit beds for high-risk EOC patients with parallel intensification of their peri-operative management by dedicated anaesthetists with a special interest in complex gynaecological oncology surgery [24]. In this respect, the years 2016–2017 served as transition years, which were further evaluated in the years 2018 and 2019.

Candidate predictors were selected a priori from three domains:

- Patient: age, year of diagnosis, year of surgery, Eastern Co-operative Oncology Group (ECOG) performance status (PS), histology type, grade (low and high), stage (FIGO 3 or 4), and pre-treatment and pre-surgery CA-125.
- Operative/tumour factors: timing of surgery (PDS or IDS), presence of ascites (yes/no), operative time, site of intra-operative bulk of the disease, size of the largest tumour deposit, Peritoneal Carcinomatosis Index (PCI), and intra-operative mapping of ovarian cancer (IMO).
- Age of consultant surgeon, as a parameter of surgical technique heuristics.

The patient- and surgery-specific variables are readily available in tertiary centres. They have been previously shown to be independent predictors of post-operative morbidity and mortality for ovarian cancer patients [3]. At the beginning of the surgery, the PCI was calculated to describe the extent of the tumour load [25]. The location of the disease was assessed using the IMO score [26]. Both PCI and IMO were calculated at laparotomy. The surgical complexity score (SCS) was assigned to describe the surgical effort according to the Aletti classification, but was interrogated as a continuous dependent variable [10]. Complete cytoreduction was defined as macroscopic tumour clearance with no residual visible disease, documented by a comprehensive visual assessment of all the areas of the abdomen.

Descriptive statistics were displayed by frequency and percentages for binary and categorical variables and by means and standard deviation (SD) or medians (with lower or upper quartiles for continuous variables). The *t* test for continuous variables and the Chi-square test for categorical variables were performed. The Fischer’s exact tests were used for binary variables. Statistical significance was set at *p* < 0.05. For these analyses, the Python’s SciPy library (version 2.7) (Python Software Foundation. Python Language Reference, version 2.7. Available at http://www.python.org) was used.

The R0 resection prediction was formulated as a binary classification problem where the positive class is related to residual disease (non R0), while the negative one is related to R0 resection. The dataset was initially split into training and test cohorts (70%:30% ratio) creating and finetuning the model with the former and evaluating it with the latter. There was no significant difference (*p* > 0.20) between the two cohorts, with respect to all variables. The training cohorts were used to create and finetune the predictive model by selecting the set of parameters that maximize model performance. Five-fold stratified cross-validation (CV) was used towards this direction. Because the dataset was imbalanced, stratified folds were created to ensure the same distribution of negative and positive classes were present in each fold, in order to reflect the distribution of the whole training dataset when performing the CV evaluation. The CV process was iterated 100 times in order to decrease both variance and bias, hence creating and evaluating 500 models in each round. Model performance was assessed by measuring the total area under the receiver-operating curve (AUC). The test set was finally employed to evaluate model’s performance with data that were not used in model creation. The eXtreme Gradient Boosting (XGBoost) method was employed to develop the predictive model. This method is an application of a generalized gradient boosting algorithm, which boosts the performance of weak learning algorithms by combining all the generated hypotheses into a single hypothesis [27]. In this work, one new weak learner was added at a time and existing weak learners in the model were frozen and left unchanged. In XGBoost, several parameters need to be selected to maximize model performance. In our case, we investigated the combined effect of eight parameters by evaluating a grid of 7680 combinations of values using Scikit-learn’s GridSearchCV function.

An explanation is that the collection of features in the interpretable domain contributed to the classification of the problem. To explain the predictive models, we proposed the Shapley Additive explanations (SHAP) values as a unified measure of feature importance [28]. This game-theory inspired method attempts to enhance interpretability by computing the important values for each feature for individual predictions. In this way, it maintains desirable properties such as local accuracy and consistency. The method explains a model globally by expressing it as a linear function of features. In other words, it explains how much the presence of a feature contributes to the model’s overall predictions.

## 3. Results

A total of 576 EOC patients with histological confirmation of EOC were initially enrolled in the study. Four patients were excluded: one patient with a synchronous primary malignancy and three patients who underwent emergency cytoreduction with no intention to treat. One patient was omitted due to incomplete data. Finally, 571 EOC patients participated in the final analysis (Table 1). The mean age of patients in the entire cohort was 63.5 + 11.2 years. This was comparable between the train and test sets; 63.6 ± 11, 63.2 ± 11.7, respectively, but CC0 was more likely to be achieved in younger patients compared to non-CC0 (*p* < 0.001). Performance status, tumour grade and stage, timing of surgery, index pre-treatment and pre-surgery CA-125, and presence of ascites intra-operatively values were similar in the groups of patients with or without CC0. As expected, real-time metric features, such as PCI, IMP, SCS, size of the large tumour bulk, and site of largest bulk were statistically different between the two groups.

### 3.1. XGBoost Model Performance

**Figure 1:**
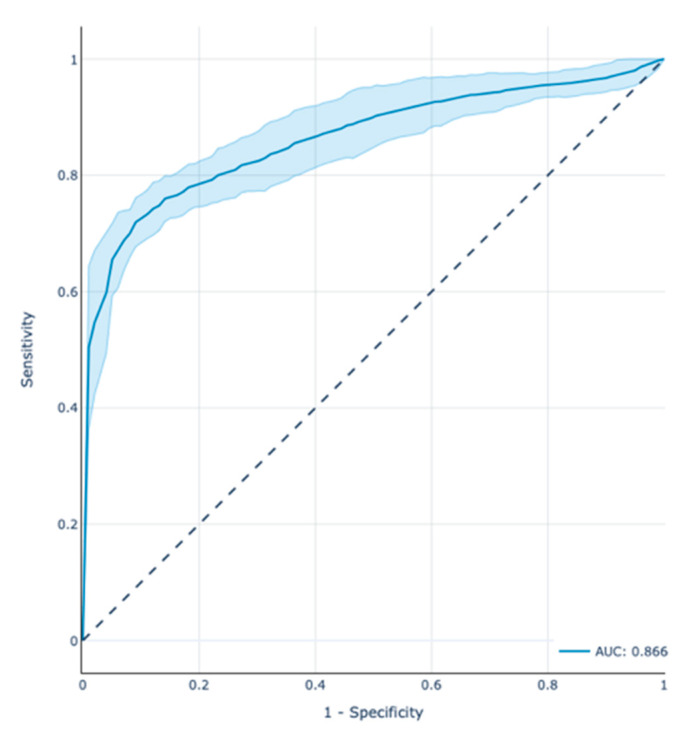
Prediction performance of the XGBoost model in the test cohort through the Receiver Operator Characteristic (ROC) (AUC = 0.866) plot demonstrating also the CI (AUC = 0.8–0.93). Note that the ROC curves depend on the order of the feature relative risk values without the need to choose a threshold.

The receiver operator characteristic area under the curve (ROC-AUC) score of the model in the test set was 0.866 with a 95% confidence interval (CI) between 0.8 and 0.93. Figure 1 depicts the ROC curve along with the CI estimated through the 100 repetitions of the CV process. Table 2 presents the results of precision, recall, and f1-score for both positive and negative classes.

To promote reproducibility, the optimal set of model parameters were the following: ‘alpha’: 0.001, ‘colsample_bytree’: 0.75, ‘learning_rate’: 0.01, ‘max_depth’: 3, ‘n_estimators’: 500, ‘scale_pos_weight’: 1.86, ‘subsample’: 0.75.

### 3.2. Feature Analysis

To demonstrate the value of our model’s explained predictions and refine features influencing CC0 resection, we developed: (a) SHAP Summary plots for global and local explanation of the results, (b) SHAP Dependence plots of the key risk features for CC0 resection, (c) SHAP Interaction Value Dependence plots, and (d) SHAP Force plots that explain the CC0 probability for individual patients.

### 3.3. SHAP Summary Plots

**Figure 2:**
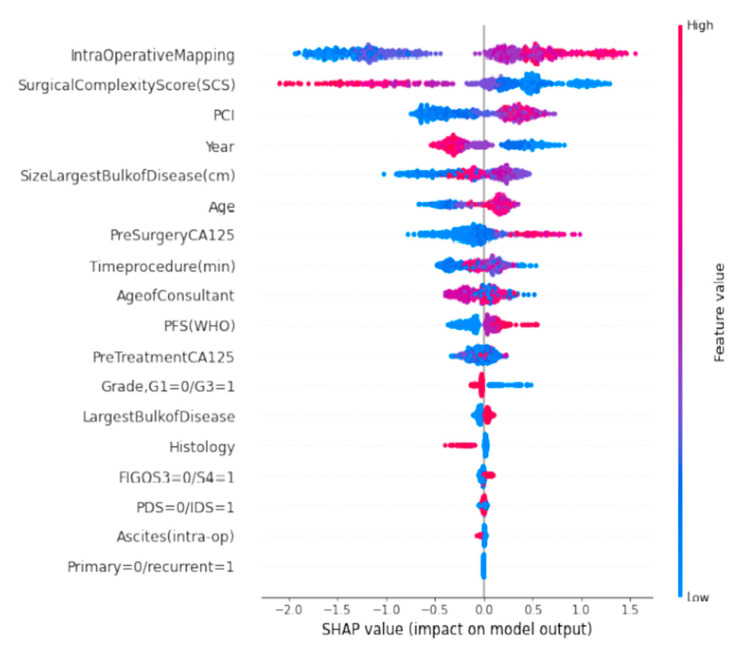
A set of beeswarm plots for global explainability of CC0 resection prediction. Dots correspond to the individual EOC patients in the study.

The SHAP Summary plot was presented in the form of a set of beeswarm plots (Figure 2). The order of the features reflects their importance, i.e., the sum of the SHAP value magnitudes across all the samples. Each point on the summary plot is a Shapley value for a feature and an instance. The position on the *y*-axis is determined by the feature and on the *x*-axis by the Shapley value. The colour represents the value of the feature from low to high. The IMO score was the most important feature globally. The plot indicated the direction of the effects, meaning, for example, that low IMO score subjects carried a higher probability of achieving CC0 than high-IMO-score subjects. In contrast, higher SHAP values of surgical complexity, the second most important feature, corresponded to a higher chance of CC0 resection. The top-six list of important features was complemented by PCI, year of surgery, and size of the largest tumour bulk. The plot also presents the distribution of effect sizes, such as the long tails of several features. These long tails suggest that features with low global importance can be equally important for specific patients. For example, CA-125 is not included in the top-six list of important features; however, in several cases, a high CA-125 value may sensibly indicate that CC0 resection is not achievable.

### 3.4. SHAP Dependence Plots

**Figure 3:**
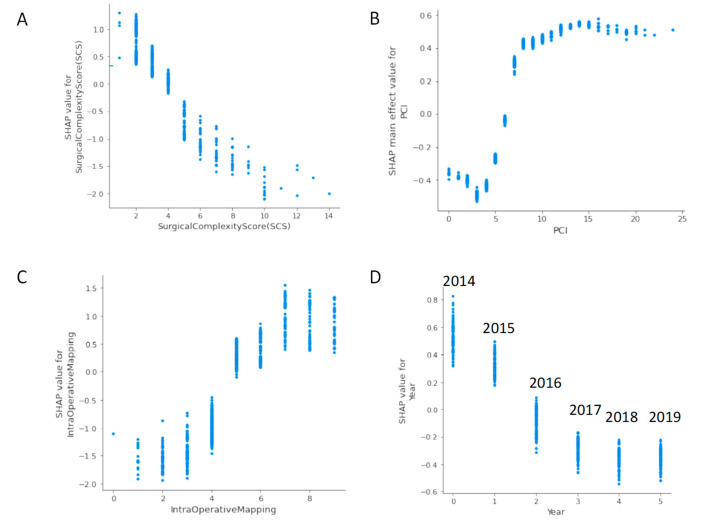
SHAP dependence plots of the top-six global explainability features versus their SHAP value for R0 resection prediction: (**A**) IMO score, (**B**) PCI, (**C**) SCS, and (**D**) year of surgery.

The SHAP dependence plot reveals the impact of each feature’s value to the prediction problem (Figure 3). It plots the value of a feature on *x*-axis and the SHAP feature value on the *y*-axis by changing each time to a specific feature in the model. Ignoring the colour of the figure, Figure 3A clearly shows the inflection point of the impact of the IMO score on the CC0. For low IMO score, overall SHAP values are negative up to five, which means that CC0 is likely to be achieved. Then, SHAP values become positive, which means that by increasing the IMO score, the probability for incomplete cytoreduction increases. Clearly, if PCI is selected as a feature to determine its impact, an increasing PCI above five results in a lower chance of CC0 (Figure 3B). Similarly, the inflection points for SCS, year of surgery, size of largest bulk of disease, age and pre-surgery CA125 are four, 2016, 5 cm, 60 years, and less than 1000, respectively (Figure 3C,D and Figure S1).

### 3.5. SHAP Interaction Value Dependence Plots

**Figure 4:**
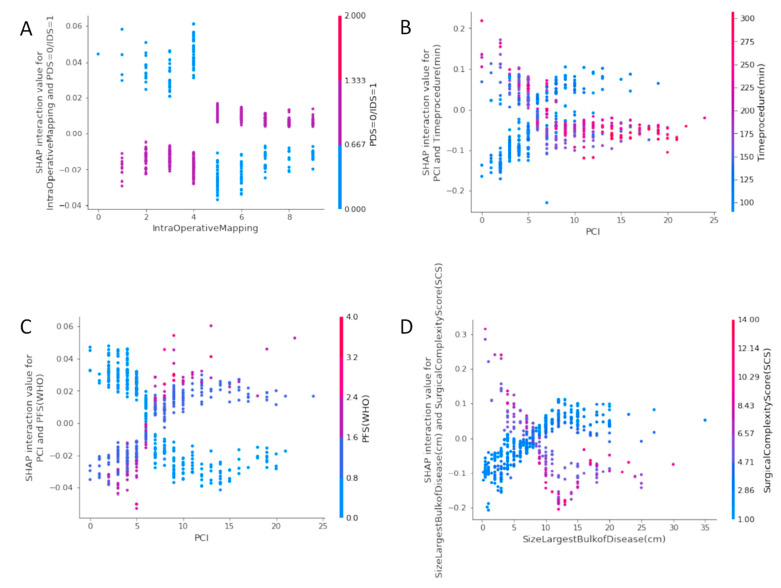
SHAP Interaction Value Dependence plots: (**A**) IMO score with type of surgery (**B**) PCI with time of the procedure (**C**) PCI with ECOG status (**D**) Size of largest tumor deposit with SCS.

The SHAP interaction values can be interpreted as the difference between the SHAP values for feature A when feature B is present, and the SHAP values for future B when feature A is absent [29]. An interaction feature is the additional combined feature effect after accounting for the individual feature effects. Examples of the plots of the SHAP interaction values of various pairs of features are shown in Figure 4.

The plot of the SHAP interaction value of the IMO score with type of surgery (Figure 4A) shows how the effect of the IMO score depends on the probability of CC0 with the type of surgery. Notably, an IMO score four or less stands a chance for CC0 at IDS, whereas, surgical effort at PDS could still achieve CC0 irrespective of how high the IMO score can be. The plot of the SHAP interaction value of PCI with time of the procedure (Figure 4B) shows that PCI has a different effect on the probability of CC0 depending on the operative team. A surgical effort up to 200 min is required to achieve CC0, provided the PCI is less than six. The plot of the SHAP interaction value of PCI with ECOG status (Figure 4C) shows that the probability for CC0 for good PS (0–1) is the same, provided the PCI is six or less. The plot of the SHAP interaction value of the size of the largest tumour bulk with SCS shows that in areas of moderate surgical effort, the effect of size on the probability of CC0 reverses at a point around 5–10 cm.

### 3.6. SHAP Force Plots

**Figure 5:**
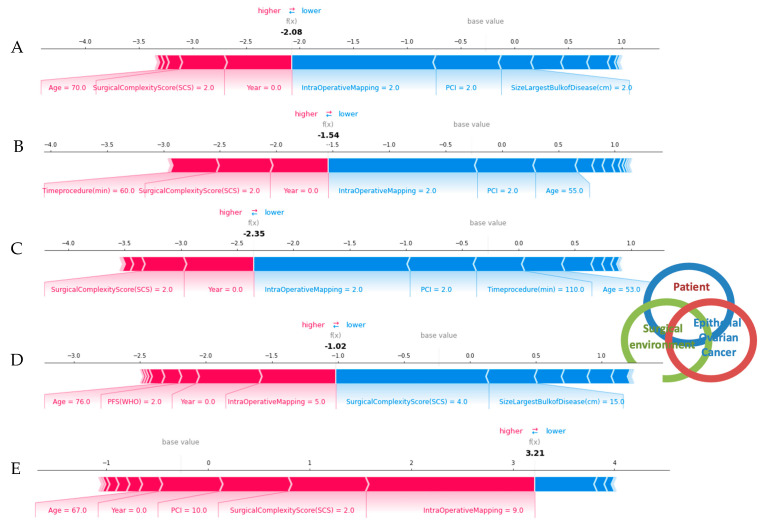
Succinct visual summary of feature integration by the XAI model into a single risk for R0 resection prediction. The SHAP force plots illustrate examples of explained risks for individual patients. For R0 resection risk, blue features have values that increased the risk, while red features decreased the risk. The combination of impacts of all features is the predicted R0 prediction risk. (**A**–**D**) The odds for R0 resection range between 1.54 and 2.35 times higher than expected (**E**). The odds for incomplete cytoreduction are 3.21 times higher than expected. Each feature impact value represents the change in risk when that feature’s value is known versus unknown. These examples clearly demonstrated the complex interactions between patients and ovarian cancer specific features in a dynamic well supported surgical environment.

For local explainability, the SHAP Force plots demonstrate features each contributing to pushing from the base value (the average model output over the training dataset) towards the model output. Features pushing the prediction higher are shown in red, whilst features pushing the prediction lower are shown in blue (Figure 5). Several examples referring to individual cases are illustrated.

## 4. Discussion

**Figure 6:**
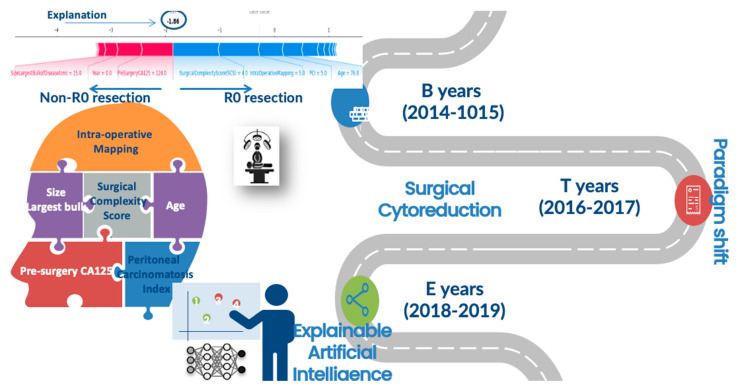
Distribution of features for global and local explainability of an AI-based model for prediction of complete cytoreduction in EOC patients. *B; baseline, T; transition, E; evaluation*.

We presented an ensemble AI-based model that predicted CC0 following cytoreductive surgery for EOC with high accuracy, and an XAI strategy that explained the patient and surgery-specific factors that led to that risk (Figure 6). To our knowledge, this is the first attempt to implement XAI models in the field of gynaecology oncology. To interpret our results, we used their visual inspection as the leading result offering constant cognitive intuition to the CC0 prediction. We surmise this is an important step towards further AI implementation in medicine because while AI models have significantly improved the ability to predict a patient’s outcome, the inability to explain the prediction from accurate, complex models remains a serious limitation. More importantly, we demonstrated how to preserve the prediction accuracy and retain interpretability by developing a method to provide theoretically justified explanations of the R0 prediction, which further builds on recent advances in the explainability of prediction methodology. This ability to provide simple explanations of predictions from an arbitrarily complex model helps eliminate the accuracy versus interpretability trade-off, thus broadening the scope of AI in the delivery of modern ovarian cancer care.

Several approaches have been proposed to explain model predictions [30]. Their scope varies based on their ability to generate global or local explanations, whereas their flexibility indicates whether their approach is model-specific or model-agnostic. Local explanations are critical because they reveal the impact of input features on individual predictions of a single patient. In our study, the SHAP approach was chosen because despite being a local explainability model, it introduces a global interpretation methodology based on the aggregations of Shapley values. Due to its ability to simplify data, such a methodology has been slowly embraced in a range of domains, including medicine [31].

This model integrated a comprehensive dataset consisting of high-fidelity, real-time data, such as IMO, PCI, operative time and surgical complexity, and static data such as patient- and tumour-specific information, resulting in a high prediction accuracy that outperforms alternative prediction models previously used to predict CC0. Using a novel XAI methodology, we explained why a prediction was made regardless of the complexity of the AI model. A numeric representation is useful (i.e., odds ratio), but a detailed explanation that the risk is due to patient’s age, IMO, SCS, PCI, maximum tumour size, and the resources provided in the dynamic surgical environment becomes more clinically meaningful since some features may be potentially modifiable and result in clinical changes mitigating that risk. Interestingly, we identified specific “turning points” that demonstrated clear preference towards CC0. For example, the strongest positive influence was exerted by IMO and PCI scores less than five and six, respectively, at the cost of a moderate surgical effort (SCS > 4). The clear-cut threshold of the IMO score—a more simplistic adaptation of the PCI—less than five has not been previously documented. However, the positive correlation between IMO and SCS has been demonstrated by London study groups [32]. The PCI score is widely perceived as an excellent predictor of incomplete cytoreduction; in that same study, NACT was considered if the PCI was higher than 24 probably due to reduced surgical success [33]. Another feature was the patient’s age. Not surprisingly, age has been frequently included in risk stratification algorithms [34]. According to Eisenkop et al., the probability of survival is independently influenced by age (≤61 years) [35]. Our cut-off of 60 years simply reflects the survival impact of surgical cytoreduction. However, we should cautiously embrace the value of features with large standard deviations, such as age and CA-125 values. Our results also showed that the optimisation of infrastructural support driven by the paradigm shift towards more complex surgical management of EOC can help achieve the goal of CC0, partly by performing more radical cytoreductive surgery for our patients, in line with other studies [36]. Reassuringly, the explanations for our predictions are broadly consistent with prior knowledge from the gynaecologic oncology community.

We deployed an efficient, theoretically justified XAI technique to estimate the importance of each feature on a prediction made for a single patient, which drives real-time explanations. This is important as it provides valuable and detailed information tailored to an individual clinical profile. The effective visualizations of such explanations could be potentially encoded in a compact visual form for gynaecologic oncologists to use. The magnitude of such a strategy can be appreciated if we extrapolate the real-time results to more than 2000 annual cytoreductive surgeries in the UK. Then, the ‘no residual disease’—a surgical note—stops being stochastic and may, in turn, boost patients’ motivation to engage in shared decision making and to act upon risk-relevant information [37].

This study further prepares the ground for a crucial full-scale clinical audit to map ovarian cancer surgery across the National Health Service. We have continuously audited and recently published our surgical outcomes. We demonstrated that improving complete surgical cytoreduction rates in advanced stage EOC is achievable without a significant increase in morbidity [15,38]. Our complete surgical cytoreduction rates were comparable to other high-volume specialized centres [39]. Standardization of surgical practice and identification of centres of excellence will benefit patients by supporting a maximal effort approach at all possible levels [40].

Several predictive models have demonstrated their ability to forecast CC0 [10,39,41,42,43]. However, some of these studies did not aim for CC0 resection, resulting in some of the results not being confirmed in cross-validation studies. The poor predictive value of cross-sectional imaging in those studies suggests that the inclusion of pre-operative radiological predictors should be used with caution, probably due to a lack of universally applicable criteria. The debate is ongoing as few groups have successfully combined pre-operative radiological and surgical observation data to construct dynamic predictive models to predict optimal or incomplete cytoreduction [44]. In our study, we did not use any radiological parameters, which could potentially explain the superior performance of our model. This observation precludes the prediction of CC0 based on the extent of tumour dissemination prior to cytoreduction. Hence, operative efforts should not be abbreviated on the hypothesis that extensive disease at specific anatomic regions precludes long-term survival [45].

A strength of the study was the development of a highly predictive model for CC0. Nevertheless, the main strength of the study was the provision of two levels of explainability for our predictive model. From a medical perspective, this is of paramount importance, as first level (global) explainability allows us to understand how the system arrived at general conclusions akin to a generic laboratory test. Furthermore, it provides second level (local) explainability that allows us to identify which features are important for the individual prediction. In other words, we explained why a certain prediction was made for a patient, i.e., specific patient characteristics that led to the prediction. In this respect, an individual CC0 prediction can be safe-checked for patterns that might indicate a false prediction, such as an outlier with an unusual feature distribution. In an era where surgeons are often subjected to scrutiny as to why they consider extending their therapeutic effort to patients with a presumed less-favourable prognosis [46], this strategy should have implications for surgeon’s acceptability, patient’s acceptance, and deserve future integration within healthcare systems’ infrastructure.

To reduce selection bias, we used strict selection criteria as per MDT’s recommendations. Our study was a single-institution experience of a tertiary referral centre, and we are conscious that our results may not be generally applicable. The applicability of these findings to other centres remain to be determined. A recent anonymized UK survey confirmed a variation in the surgical management of EOC amongst consultant gynaecologic oncologists. Based on the mean operating times and types of procedures undertaken, the survey provided compelling evidence that in many UK cancer centres, the surgical goal has not been complete cytoreduction [47]. The retrospective study nature could not necessarily be a limiting factor, as surgical efforts are often interpretable in the sense that they can only be rationalized after surgery. Static features, such as BMI or co-morbidities, frequently used as a proxy for patient acuity, have been employed in our previous effort to predict CC0 in an earlier subset of this study population [14]. They did not appear neither significant nor of relative importance when building the AI model. Temporal recovery-related features may be more insightful than those derived from measures of disease severity at a single point in time [48]. Another limitation is the lack of prospective quality-of-life data. Ongoing research aims to implement an ensemble AI-based model to predict surgical complications following EOC cytoreductive surgery, an important short-term outcome. However, standard machine learning algorithms perform rather poorly on such datasets as they tend to be biased towards the majority class. This decreases the prediction accuracy of the minority class, i.e., the small high-risk group with surgical complications [49]. We also acknowledge the fact that we did not use survival as an outcome, predominantly focusing only on the advanced-stage EOC patients who received surgery. This might potentially introduce selection bias when evaluating survival, as the denominator should include all presenting EOC patients.

## 5. Conclusions

We developed a highly accurate AI-based model for CC0 prediction following EOC cytoreductive surgery. We provided novel global and local feature set explainability that can be used for quality control and internal audits. The study showcases the strong need for explainable AI; as we unveil the black box of AI, we remove barriers to its use in clinical practice, thereby allowing the application of AI in modern ovarian cancer care.

## Figures and Tables

**Table 1:** Cohort descriptive statistics: values are mean ± SD or *n* (%).

Demographic Characteristics	Overall (*n* = 571)	Train Set (*n* = 399)	Test Set (*n* = 172)	*p* Value	Non R0 (*n* = 196)	R0 (*n* = 375)	*p* Value
Age (y)	63.5 ± 11.2	63.6 ± 11	63.2 ± 11.7	0.71	65.6 ± 10.1	62.4 ± 11.6	**<0.001**
Histology				0.217			**0.008**
Serous	504 (0.88)	350 (0.88)	153 (0.89)		186 (0.94)	318 (0.85)	
Mucinous	13 (0.02)	7 (0.017)	6 (0.035)		1 (0.005)	12 (0.03)	
Clear cell/endometrioid	33 (0.06)	23 (0.057)	10 (0.058)		6 (0.03)	27 (0.07)	
Miscellaneous	22 (0.04)	19 (0.047)	3 (0.017)		4 (0.02)	18 (0.05)	
Primary = 0/recurrent = 1							0.37
Primary	561 (0.98)	370 (0.66)	191 (0.32)	0.7	192 (0.98)	369 (0.98)	
Recurrent	10 (0.02)	7 (0.02)	3 (0.02)				
Grade (G1 = 0/G3 = 1)	516 (0.9)	354 (0.89)	162 (0.94)		174 (0.89)	342 (0.91)	0.43
FIGO stage (S3 = 0/S4 = 1)	157 (0.27)	106 (0.27)	51 (0.3)		59 (0.3)	98 (0.26)	0.36
Performance status (WHO)				0.74			**0.001**
0	273 (0.48)	192 (0.48)	80 (0.46)		70 (0.35)	203 (0.54)	
1	212 (0.37)	144 (0.36)	68 (0.39)		90 (0.46)	122 (0.32)	
2	68 (0.12)	50 (0.125)	18 (0.1)		28 (0.14)	40 (0.1)	
3	19 (0.03)	13 (0.276)	6 (0.35)		9 (0.04)	10 (0.02)	
Age of consultant (y)	49.2 ± 6	49.3 ± 6	48.9 ± 6	0.46	49.2 ± 6.3	49.2 ± 5.9	0.99
Timing of surgery				0.6			0.38
Interval debulking surgery	396 (0.69)	278 (0.7)	118 (0.69)		141 (0.72)	255 (0.68)	
Primary debulking surgery	175 (0.31)	113 (0.64)	62 (0.36)		122 (0.7)	53 (0.3)	
Year				0.7			**0.012**
2014	91 (0.158)	63 (0.158)	27 (0.156)		44 (0.22)	47 (0.12)	
2015	93 (0.162)	65 (0.163)	28 (0.16)		36 (0.18)	57 (0.15)	
2016	108 (0.19)	74 (0.185)	34 (0.198)		38 (0.19)	70 (0.19)	
2017	96 (0.17)	64 (0.16)	32 (0.186)		31 (0.16)	65 (0.17)	
2018	82 (0.14)	55 (0.138)	27 (0.157)		23 (0.12)	59 (0.16)	
2019	102 (0.18)	78 (0.195)	24 (0.14)		25 (0.13)	77 (0.21)	
Pre-treatment CA125	1572.7 ± 2993.2	1542.9 ± 3070.7	1641.8 ± 2812.9	0.7	1790.9 ± 3207	1458.7 ± 2873.1	0.22
Pre-surgery CA125	411.79 ± 1170	387.3 ± 943.7	468.6 ± 1576.7	0.52	451 ± 931.7	391.3 ± 1277.7	0.52
Size of largest disease bulk (cm)	8.9 ± 5.58	9.0 ± 5.79	8.63 ± 5.09	0.43	9.79 ± 5.24	8.41 ± 5.7	**0.004**
Peritoneal Carcinomatosis Index (PCI)	7.39 ± 4.53	7.39 ± 4.52	7.4 ± 4.56	0.99	8.91 ± 4.31	6.6 ± 4.44	**<0.001**
Surgical Complexity Score (SCS)	3.8 ± 2.12	3.79 ± 2.14	3.82 ± 2.09	0.89	3.1 ± 1.44	4.17 ± 2.32	**<0.001**
Time procedure (min)	170.36 ± 77.48	170.1 ± 79.48	170.96 ± 72.85	0.9	161.84 ± 63.81	174.81 ± 83.47	0.039
Site of largest tumour deposit				0.28			**<0.001**
Ovary	298 (0.52)	216 (0.54)	82 (0.477)		83 (0.42)	215 (0.57)	
Omentum	258 (0.45)	171 (0.43)	86 (0.5)		110 (0.56)	148 (0.39)	
Miscellaneous	16 (0.03)	12 (0.03)	4 (0.023)		4 (0.02)	12 (0.03)	
Intra Operative Mapping Score	4.92 ± 1.99	4.95 ± 1.99	4.85 ± 2.01	0.58	5.96 ± 1.69	4.38 ± 1.93	**<0.001**
Ascites (intra-op)	131 (0.23)	93 (0.23)	38 (0.22)		49 (0.25)	82 (0.22)	0.458

**Table 2:** Performance evaluation scores of the test cohort.

	Precision	Recall	f1-Score
CC0	0.91	0.87	0.89
Non-CC0	0.71	0.78	0.75

## Data Availability

The data presented in this study are available upon request from the corresponding author.

## Supplementary Materials

The following supporting information can be downloaded at: https://www.mdpi.com/article/10.3390/jpm12040607/s1, Figure S1: Examples of SHAP dependence plots of top-six global explainability features versus their SHAP value for R0 resection prediction: (a) size of largest bulk of disease, (b) age, (c) pre-surgery CA-125.
