# Supplementary figures and images for: Explainable Artificial Intelligence for Prediction of Complete Surgical Cytoreduction in Advanced-Stage Epithelial Ovarian Cancer
Source: J Pers Med. 2022 Apr 10;12(4):607. doi: 10.3390/jpm12040607 (PMC9030484; doi:10.3390/jpm12040607)

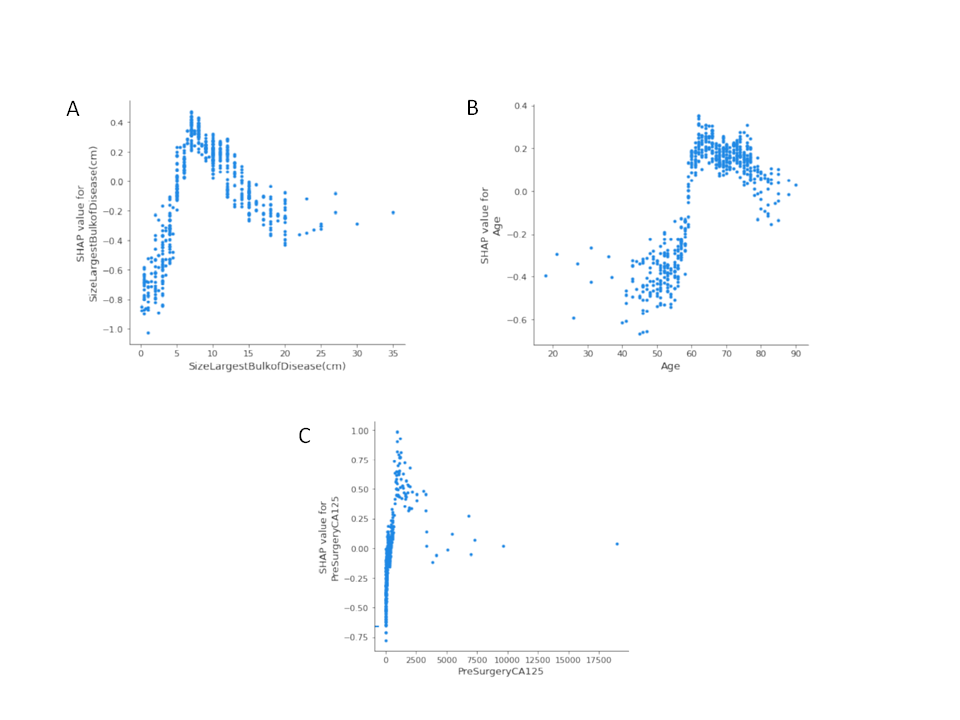

Supplement: Supplementary file 1 [file jpm-12-00607-s001.zip › jpm-1630025-supplementary.tif]
